# High intake of n-6 polyunsaturated fatty acid exacerbates non-alcoholic steatohepatitis by the involvement of multiple metabolic pathways

**DOI:** 10.3389/fnut.2025.1562509

**Published:** 2025-06-23

**Authors:** Jian-Tong, Meng-Ting Zhou, Xiang-Zhun Song, Liu Yang, Yu-Hui Fang, Lan Liu, Jing-Shu Cui, Xiao-Chen Lu, Hai-Yang Zhu, Ying-Bin Jin, Hong-Mei Han

**Affiliations:** ^1^Department of Gastroenterology, Affiliated Hospital of Yanbian University, Yanji, China; ^2^Department of Gastroenterology, Yanji Hospital, Yanji, China; ^3^Department of Gastroenterology, Jilin Provincial People’s Hospital, Changchun, China; ^4^Department of Gastroenterology and Hepatology, Characteristic Medical Center of the Chinese People’s Armed Police Force, Tianjin Key Laboratory of Hepatopancreatic Fibrosis and Molecular Diagnosis & Treatment, Tianjin, China; ^5^Department of Dermatology, Fuyang People's Hospital of Anhui Medical University, Fuyang, China; ^6^Department of Pathology, Affiliated Hospital of Yanbian University, Yanji, China; ^7^Department of Gastroenterology, Jimo District People's Hospital, Jimo, China

**Keywords:** n-6 polyunsaturated fatty acids, non-alcoholic steatohepatitis, choline deficiency, arachidonic acid, leukotrienes, lipoxygenase pathway

## Abstract

Non-alcoholic steatohepatitis (NASH) is characterized by steatosis, inflammation, and hepatocyte damage. A Western-style diet characterized by excessive n-6 polyunsaturated fatty acid (n-6 PUFA) intake, which is metabolized to pro-inflammatory arachidonic acids (AAs), might contribute to the exacerbation of NASH. Investigating the interactive effects of choline deficiency and n-6 PUFA supplementation on NASH progression, we aimed to elucidate how AA metabolites, such as leukotrienes, prostaglandins, and the CYP2J3/epoxyeicosatrienoic acids (EET) pathway influence disease pathogenesis. Rats were fed one of four diets: choline-sufficient with low n-6 PUFA and high saturated fatty acid (SFA) (C1), choline-sufficient with high n-6 PUFA (C2), choline-deficient with high n-6 PUFA (D1), or choline-deficient with low n-6 PUFA and high SFA (D2). Liver damage, inflammation, and oxidative stress in D1 were more than compared to C2 and D2 groups. Aggravation of NASH in D1 was accompanied by reduced levels of 15-deoxy-Δ12,14-prostaglandin J2 and PPAR-γ, weakening anti-inflammatory effects and lipid metabolism. Decreased CYP2J3 expression along with reduced PPAR-α levels, likely contributed to reduced anti-inflammatory EET levels, while elevated soluble epoxide hydrolase increased pro-inflammatory dihydroxyeicosatrienoic acids. Additionally, higher leukotriene C4 and 15-hydroxyeicosatetraenoic acid levels via the lipoxygenase pathway exacerbated inflammation. The combined pathway alterations in the D1 group increased inflammation, leading to elevated NF-κB expression, Kupffer cell polarization to M1, and lipid peroxidation, with n-6 PUFA interacting with choline deficiency to exacerbate these effects. Correlational analysis revealed significant associations between these pathways and inflammatory/oxidative markers. Our findings suggest that high intake of n-6 PUFA could aggravate NASH.

## Introduction

Western diets typically exhibit high n-6 PUFA intake, and emerging evidence suggests that excessive n-6 PUFA consumption may exacerbate liver inflammation and damage, and has been linked to an increased risk of non-alcoholic steatohepatitis (NASH). The prevailing high n-6 PUFA intake in Western diets underscores the urgency to investigate its impact on NASH progression. Mechanisms by which n-6 PUFA excess contributes to liver damage remains unclear.

Choline is essential for the structure of cell and mitochondrial membranes and plays a key role in liver lipid metabolism. A choline-deficient diet is known to induce steatosis, lipid peroxidation, inflammation, and liver fibrosis, characteristics of NASH. This study uses the term NASH, as the choline deficient diet model used in this study recapitulates histological features of non-alcoholic steatohepatitis [steatosis, inflammation, hepatocyte injury (1–3)] though it does not fully replicate the metabolic syndrome aspects of human metabolic dysfunction-associated steatohepatitis (MASH). Moreover, excessive n-6 PUFA administration has been shown to worsen liver damage in animal models (1, 2, 4, 5). While combining choline deficiency with methionine deficiency (MCD) synergistically exacerbates NASH in animal models, in this study, we opted to utilize a choline-deficient diet alone to study the impact of excess n-6 PUFA on NASH progression. This approach allowed us to specifically investigate the aggravating effects of n-6 PUFA on liver lipid peroxidation and inflammation, without the confounding effects of methionine deficiency, which accelerates NASH progression. By using the choline-deficient diet, we can examine the interactions between n-6 PUFA supplementation and choline deficiency in a more controlled manner. Our study aims to elucidate the mechanisms underlying the detrimental effects of n-6 PUFA on NASH.

n-6 PUFAs are metabolized into AA, a key lipid mediator in NASH. AA contributes to lipid peroxidation and inflammation as it serves as a precursor for biologically active lipids produced via three major metabolic pathways (6). First, the cytochrome (CYP) pathway, which includes both the CYP450 and the ω-hydroxylation pathways. AA is metabolized to biologically-active epoxyeicosatrienoic acids (EETs) by CYP2J through the epoxygenase pathway; EETs are then metabolized into dihydroxyeicosatrienoic acids (DHETs) by soluble epoxide hydrolase (sEH). AA can also be metabolized into 20-hydroxyeicosatetraenoic acid (20-HETE) by ω-hydroxylase. Second, the lipoxygenase (LO) pathway, which includes the 5-LO pathway, the 12-LO pathway, and the 15-LO pathway. AA is metabolized into 5-hydroxyeicosatetraenoic acid (5-HETE) and leukotriene A4 (LTA4) through the 5-LO pathway, and LTA4 is further metabolized into LTC4 by LTC4 synthase (LTC4S). AA is metabolized by 12-LO into 12-HETE and by 15-LO into 15-HETE. Lastly, the cyclooxygenase (COX) pathway metabolizes AA into prostaglandins and thromboxanes (7, 8). The metabolism of AA via the CYP P450, LO, and COX pathways produces various bioactive lipids with counterregulatory effects on NASH progression.

Choline deficiency causes significant changes in AA metabolites, which have been implicated in NASH pathogenesis (9, 10). Since n-6 PUFA are the precursors of AA, we surmised that choline deficiency may interact with n-6 PUFA. In the present study, we used rats with NASH induced by a choline-deficient diet that were given excess n-6 PUFA to understand the relationship between choline deficiency and n-6 PUFA. Specifically, we aimed to determine whether n-6 PUFA, in combination with choline deficiency, aggravate hepatic lipid peroxidation and inflammation in NASH by altering the AA metabolic pathways. To achieve this goal, we employed a multidisciplinary approach, combining histological, biochemical, and molecular analyses with correlation studies, to investigate how n-6 PUFA and choline deficiency synergistically worsen NASH via CYP450, LO, and COX pathways, thereby bridging gaps in our understanding of dietary interactions.

## Materials and methods

### Materials

Choline-sufficient, amino acid-defined [(CSAA), (Cat# TP 01010GS)] diet and choline-deficient, amino acid-defined [(CDAA) diet (Cat# TP 01010G)] were procured from Nantong Trophic Animal Feed High-Tech Company, China. Nutrition composition of the diets is provided in Supplementary Table S1. BCA protein quantitation kit (Boster Bio, Cat # AR0146), RIPA lysis buffer (Boster Bio, Cat # 0105), protease inhibitor cocktails (Boster, Bio Cat # AR1182), phosphatase inhibitor (Boster Bio, Cat # AR1183), color pre-dyed protein marker (Boster Bio, Cat # AR1113), Western-specific primary and secondary antibody diluent (Boster Bio, Cat # AR1017), wash buffer TBS-T (Boster Bio, Cat # AR0195-10), ECL chemiluminescent reagent (Boster Bio, Cat # AR1196), BSA TBS buffer system blocking solution (Boster Bio, Cat # AR0189), NF-κB antibody (Boster Bio, Cat # A01228-1), GAPDH antibody (Boster Bio, Cat # M00227), HRP-conjugated goat anti-rabbit IgG (Boster Bio, Cat # BA1054), CD163 antibody (Boster Bio, Cat # A00812-2), CD11c antibody (Boster Bio, Cat # A00357-3), CD68 antibody (Boster Bio, Cat # BA3638), fluorescent (DyLight 488) labeled goat anti-rabbit IgG (Boster Bio, Cat # BA1127), fluorescent (DyLight 594) labeled goat anti-rabbit IgG (Boster Bio, Cat # BA1142), rat TNF-α ELISA kit (Jiangsu Enzyme Immunoassay Co., Ltd., Cat # MM-0180R1), rat IL-1β ELISA kit (Boster Bio, Cat # EK0393), rat IL-4 ELISA kit (Jiangsu Enzyme Immunoassay Co., Ltd., Cat # MM-0191R1), rat IL-10 ELISA kit (Boster Bio, Cat # EK0418), 25 VD3 assay kit (Roche Diagnostics, Cat # 07028148190), TAOC assay kit (Nanjing Jiancheng, Cat # A015-3-1), MDA assay kit (Nanjing Jiancheng, Cat # A003-1-2), and free fatty acid kit (Kunchuang Biotechnology, Xian, China, Cat # SK125-2).

### Animal model treatments

All animal experimental procedures followed the Ethical Guidelines of the China Association of Laboratory Animal Care and were approved by the Ethics Committee of Yanbian University (Approval number: YD20231027006). Twenty-four specific-pathogen-free-grade, male Wistar rats (6 weeks old, weighing 180–200 g) were obtained from the Yisi Experimental Animal Technology Company (Changchun, China); two rats were housed together in one cage. All rats were adapted to controlled conditions of a 12 h light/dark cycle, a temperature of 22 ± 2°C, and a humidity of 40 ~ 60%. All rats had free access to sterile water and rat pellet diet procured from Trophic Animal Feed High-Tech Co., Ltd. (Jiangsu, China).

#### Experimental groups

**Figure 1:**
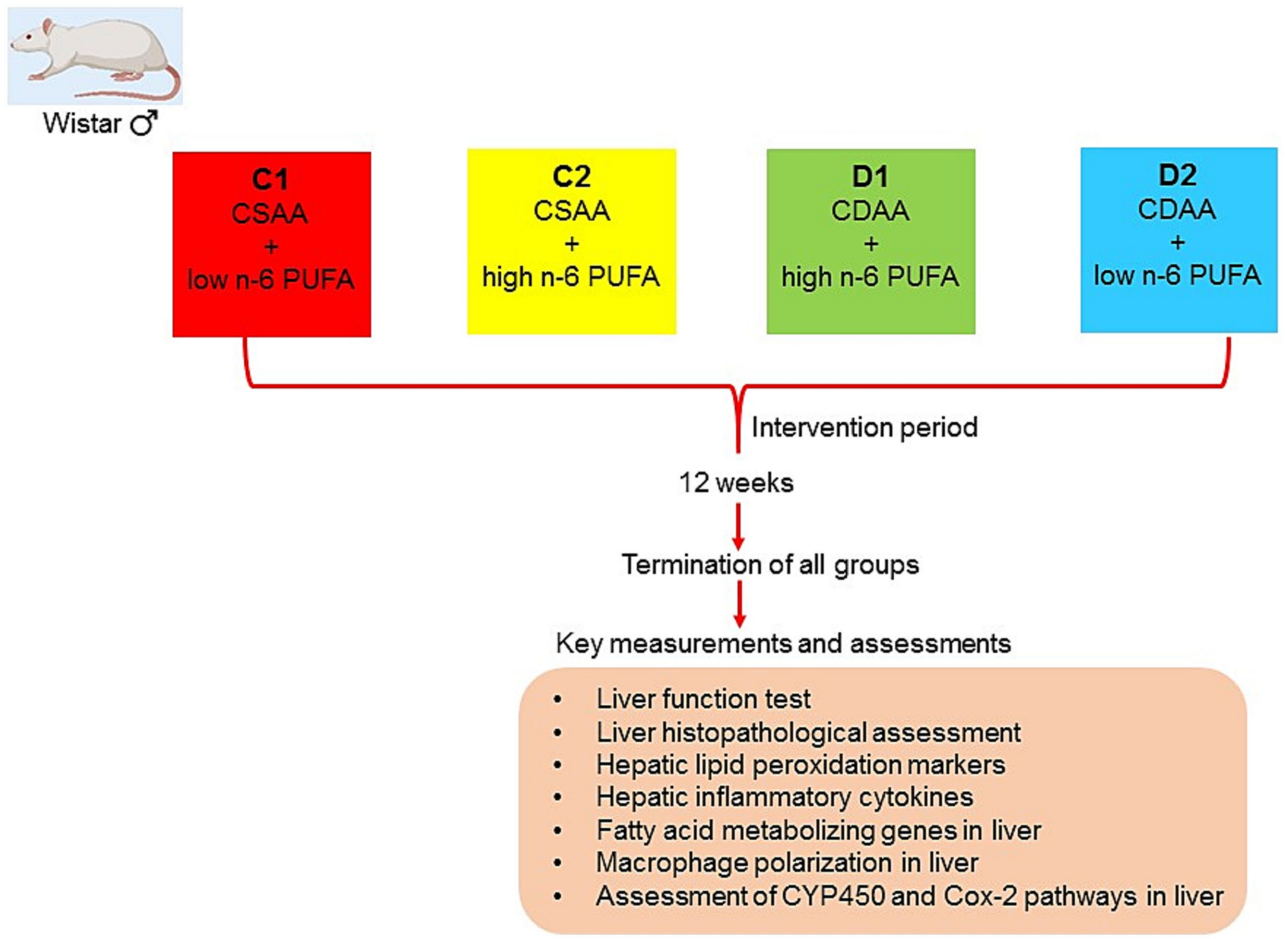
A schematic diagram representing study design. Diet compositions are detailed in Supplementary Table S1. The study was approved by the Ethics Committee of Yanbian University (Approval number: YD20231027006). Further details can be found in the Materials and Methods section, under Animal Treatments.

After 1 week of normal feeding for adaptation, rats were randomly assigned to one of four groups: (1) a CSAA diet with low n-6 PUFA and high saturated fatty acid (SFA), where 10 g of coconut oil was added to every 100 g of this diet (C1, *n* = 6) (11); (2) a CSAA diet with high n-6 PUFA, where 10 g of corn oil was added to every 100 g of this diet (C2, *n* = 6) (1, 12); (3) a CDAA diet with high n-6 PUFA, where 10 g of corn oil was added to every 100 g of this diet (D1, *n* = 6); and (4) a CDAA diet with low n-6 PUFA and high SFA, where 10 g of corn oil was added to every 100 g of this diet (D2, *n* = 6). Figure 1 presents the schematic diagram of the experimental design and Supplementary Table S1 provides composition of the diets used in the study.

#### Rationale for the experimental groups

C1 group received a choline-sufficient diet with SFA and low n-6 PUFA, and served as the control. This allowed for comparison with other groups to evaluate the effects of choline sufficiency and SFA on liver health. C2 group helped determine how high n-6 PUFA affected liver health in the presence of adequate choline, serving as a control to compare against the effects of choline deficiency. The D1 group, on a choline-deficient diet with high n-6 PUFA, was used to evaluate how elevated n-6 PUFA levels aggravate NASH progression under choline deficiency. D2 group received a choline-deficient diet with SFA and low n-6 PUFA to induce NASH, enabling evaluation of how SFA affects NASH development relative to other groups. Together, this study design helped evaluate how choline deficiency alone impacted liver health and how it interacted with a high-SFA diet.

Comparisons between these groups aimed to determine: (1) the impact of high n-6 PUFA on liver health with adequate choline (C1 vs. C2), and (2) the influence of choline deficiency on liver health with high n-6 PUFA (D1 vs. D2).

#### Sample harvesting from the experimental groups

After 12 weeks of treatments as described above, the rats were starved for 12 h, and then euthanized by cervical vertebrae dislocation. The weight of the rats was measured and blood was collected from the abdominal aorta; serum was separated, and stored at −80°C until further use. The liver was carefully separated, the wet weight was measured, and a piece of tissue from the middle of the right lobe was fixed in 4% formaldehyde solution for preparation of paraffin sections. The remaining liver samples were homogenized, which was immediately frozen in liquid nitrogen and stored at −80°C until further use.

### Liver index

The liver index was calculated as a percentage as follows:

[Wet liver weight (g)/body mass (g)] × 100.

### Evaluation of liver histopathology

Liver tissue samples were fixed with 4% formaldehyde solution for 24 h and stained with H&E. Images were captured using a light microscope at a magnification of 200×. The severity of hepatic histopathology was evaluated by steatosis, activity, and fibrosis (SAF) score. The SAF score was determined according to the criteria proposed by the NASH Clinical Research Network (13) for grading steatosis and activity (Table 1). All sections were independently graded by two pathologists blinded to the experimental design. Five random visual fields were observed and scored, with the mean value used as the final data point.

**Table 1:** NASH scoring criteria.

Steatosis grade	Percentage of hepatocytes with lipid droplets	Description
0	<5%	Negligible steatosis
1	5 to 33%	Slight steatosis
2	34 to 66%	Intermediate steatosis
3	>66%	Extensive steatosis

Lobular inflammation score	Inflammatory foci per 200x field	Description
0	None observed	Absence of inflammatory foci
1	Fewer than 2 foci	Minimal inflammatory foci
2	2 to 4 foci	Moderate inflammatory foci
3	More than 4 foci	Numerous inflammatory foci

Ballooning score	Description	Observation
0	No ballooned hepatocytes	None detected
1	Occasional ballooned hepatocytes	Limited presence
2	Abundant or markedly ballooned hepatocytes	Significant or pronounced presence

### Biochemical analyses

Serum alanine transaminase (ALT), aspartate transaminase (AST), triglyceride (TG), total cholesterol (TC), high-density lipoprotein-cholesterol (HDL-C), and low-density lipoprotein-cholesterol (LDL-C) levels were determined by a Roche cobas c702 automatic biochemical analyzer.

The levels of malondialdehyde (MDA) (A003-1-2, Nanjing Jiancheng Bioengineering Institute, Nanjing, China), interleukin-10 (IL-10) (EK0418, Boster Bio-Engineering Limited Company, Wuhan, China), tumor necrosis factor-α (TNF-α), IL-1β, and IL-4 (MM-0180R1, MM-0047R1, MM-0191R1, respectively, Jiangsu Meimian Industrial Co. Ltd., Jiangsu, China) in the liver homogenate of rat liver were determined by ELISA. The levels of LTC4 and LTC4S in liver homogenate were also determined by ELISA (MM-0392R1, MM-71776R1, respectively, Jiangsu Meimian Industrial Co. Ltd., Jiangsu, China).

### Real-time quantitative PCR (qPCR)

The mRNA expression levels of peroxisome proliferator-activated receptor-α (*PPAR-α*), *PPAR-γ1* and *Cyp2j3* in liver homogenate were determined by qPCR. Liver samples were homogenized in TRIzol, the RNA was extracted according to the manufacturer’s protocol, and RevertAid Reverse Transcriptase (EP0442, Thermo Scientific, United States) was used for reverse transcription. The THUNDERBIRD® qPCR Mixkit (QPS-201, TOYOBO, Japan) was used for qPCR. The data were processed by the ΔΔCT method. Primer sequences are shown in Table 2.

**Table 2:** Primer sequences.

Gene	Sequence (5’to 3′)	bp	Ref. Seq
PPAR-α	F: CGGGTCATACTCGCAGGAAAG	155	NM_013196.2
	R: TGGCAGCAGTGGAAGAATCG		
PPAR-γ2	F: CCTTTACCACGGTTGATTTCTC	141	NM_013124.3
	R: CAGGCTCTACTTTGATCGCACT		
Cyp2j3	F: TCAGAATGTCCGTCACCATT	75	NM_175766
	R: TTCCTCTTCGACATCACAGC		
GAPDH	F: TTCAACGGCACAGTCAAGG	114	NM_017008.4
	R: CTCAGCACCAGCATCACC		

### Western blotting

Nuclear factor-κB (NF-κB) levels in liver homogenates were measured using Western blotting. We used a primary NF-κB antibody (A00284-1, Boster Bio-Engineering Limited, Wuhan, China) at a 1:1000 dilution and rat β-actin as the housekeeping protein with a 1:2000 diluted antibody (BM0627, Boster Bio-Engineering Limited, Wuhan, China). Secondary antibodies, HRP-goat anti-rabbit and HRP-goat anti-mouse (Boster Bio-Engineering Limited, Wuhan, China), were applied at a 1:5000 dilution. Protein bands were detected using the ECL chemiluminescence reagent kit (AR1196, Boster Bio-Engineering Limited, Wuhan, China), and images were captured with an imaging analyzer. Band intensities were quantified through densitometry using ImageJ software.

### Immunofluorescent staining

Kupffer cell (KC) markers CD11c (M1-type KCs) and CD163 (M2-type KCs) in liver homogenates were measured using a dual immunofluorescence double-labeling method. Liver tissue sections were dewaxed, rehydrated, and stained for CD68 + CD11c (M1-type KCs) and CD68 + CD163 (M2-type KCs). Primary and secondary antibodies labeled with DyLight 488 and DyLight 594 fluorescein were used. After staining, sections were counterstained with DAPI and mounted with an anti-fluorescence quenching tablet. Images were captured using a fluorescence microscope (BX51, Olympus, Japan) or scanner (APERIO VERSA 8, Leica, Germany).

### Liquid chromatography–tandem mass spectrometry (LC–MS/MS)

The levels of the CYP pathway metabolites EETs, DHETs and 20-HETE; the LO pathway metabolites 5-HETE, 12HETE, 15-HETE; and the COX pathway metabolites prostaglandin E2 (PGE2), prostaglandin D2 (PGD2), prostaglandin F2α (PGF2α), and 15d-deoxyprostaglandin J2 (15d-PGJ2) in liver homogenate were determined by LC–MS/MS.

Briefly, PBS containing 0.1% (w/v) butylated hydroxytoluene and the isotope internal standard were added to the sample, incubated at 4°C for 1 h, centrifuged, and the supernatant was extracted. The supernatant was enriched with AA using an Oasis MAX SPE column. Quantitative analysis of eicosanoids after solid-phase extraction-enrichment was carried out in the electrospray ionization mode using the Exit UPLC-QTRAP 6500 PLUS (Scienx) LC–MS/MS instrument.

### Statistical analyses

The experiments were conducted independently in triplicate. Data analysis was performed using IBM SPSS Statistics 26.0, and results are presented as mean ± standard error of the mean (SEM). A two-way analysis of variance (ANOVA) was used to analyze group effects. When a significant effect was found, Fisher’s Least Significant Difference test was used as a post-hoc method to compare differences between groups. An *α* = 0.05 and *p* < 0.05 was considered statistically significant. Pearson’s correlation coefficient (*r*) was used for correlation analysis, where *p* < 0.05 was considered statistically significant. Bar charts were generated using GraphPad Prism 8.0.2.

## Results

### Impact on body mass, liver weight, index, and enzyme levels

**Figure 2:**
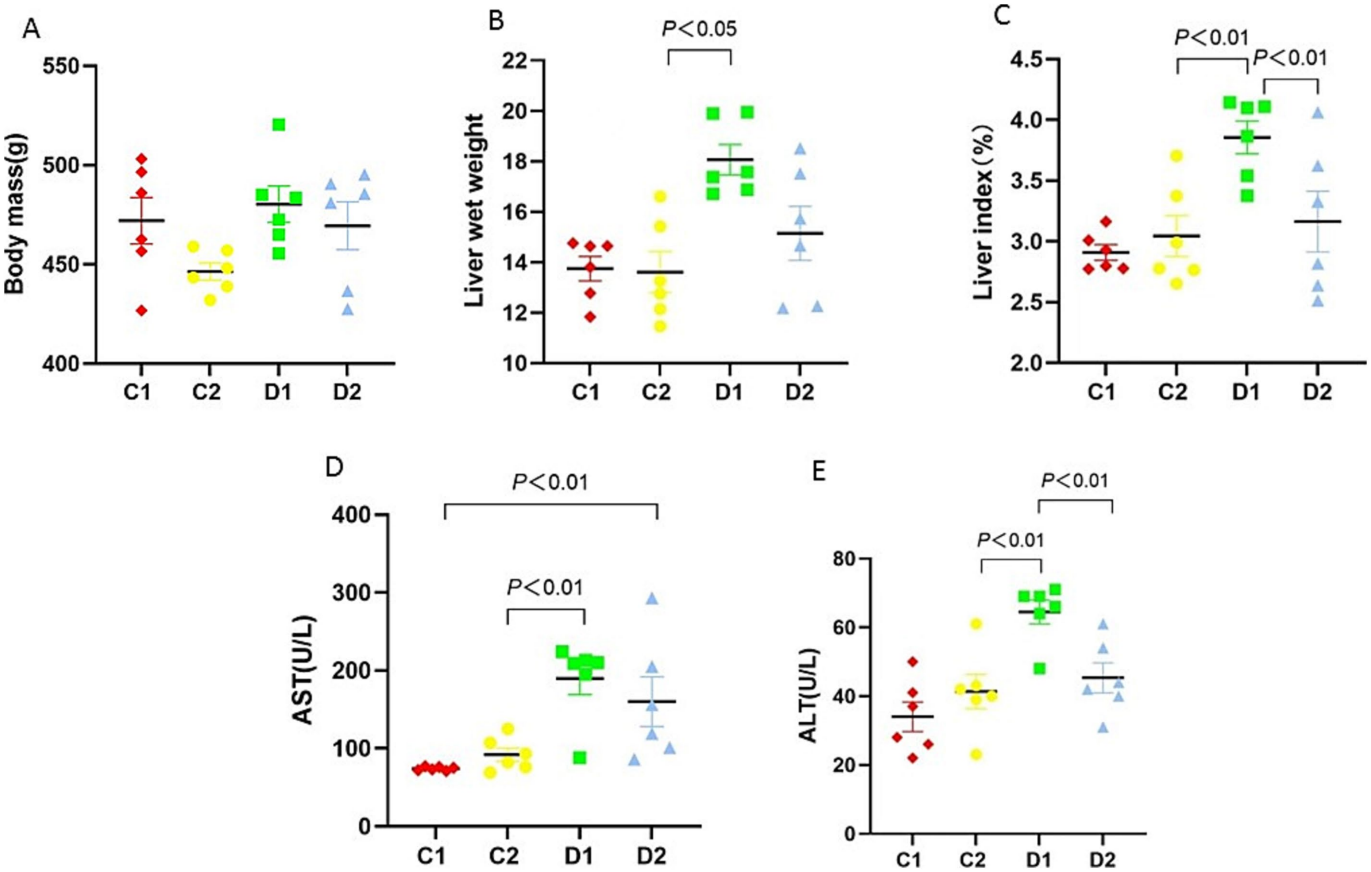
Effects of n-6 PUFA on liver index, wet weight, and biochemical markers in rats with NASH induced by a choline-deficient diet. **(A)** Body mass, **(B)** liver wet weight, **(C)** liver index, **(D)** serum AST, and **(E)** ALT levels. Data are expressed as mean ±SEM (*n* = 6/group).

To investigate the impact of choline deficiency and n-6 PUFA supplementation on liver pathology, we assessed liver wet weights and liver index in rats fed different dietary regimens. Body mass at the end of all treatments remained unchanged across all experimental groups (Figure 2A). D1 group had significantly higher liver wet weights than C2 group (18.06 ± 0.60 g vs. 13.61 ± 0.81 g, *p* < 0.01) (Figure 2B). The liver index (defined as the ratio of liver weight to body mass) of D1 group was significantly higher than that of C2 group and D2 group (3.86 ± 0.13% vs. 3.04 ± 0.17% and 3.16 ± 0.25%, both *p* < 0.01) (Figure 2C).

Liver enzyme levels are sensitive indicators of hepatic damage. We therefore evaluated the effects of choline deficiency and n-6 PUFA supplementation on serum AST and ALT activities. Serum AST in D2 group was significantly higher than in C1 group (160.00 ± 31.82 U/L vs.74.00 ± 0.97 U/L, *p* < 0.01). In addition, serum AST in D1 group was significantly higher than in C2 group (189.00 ± 20.72 U/L vs. 92.00 ± 8.56 U/L, *p* < 0.01) (Figure 2D). Serum ALT was significantly elevated in the D1 group compared to C2 and D2 groups (64.50 ± 3.45 U/L vs. 41.33 ± 4.94 U/L and 45.33 ± 4.35 U/L, both *p* < 0.01) (Figure 2E).

### Impact on liver histopathological characteristics

**Figure 3:**
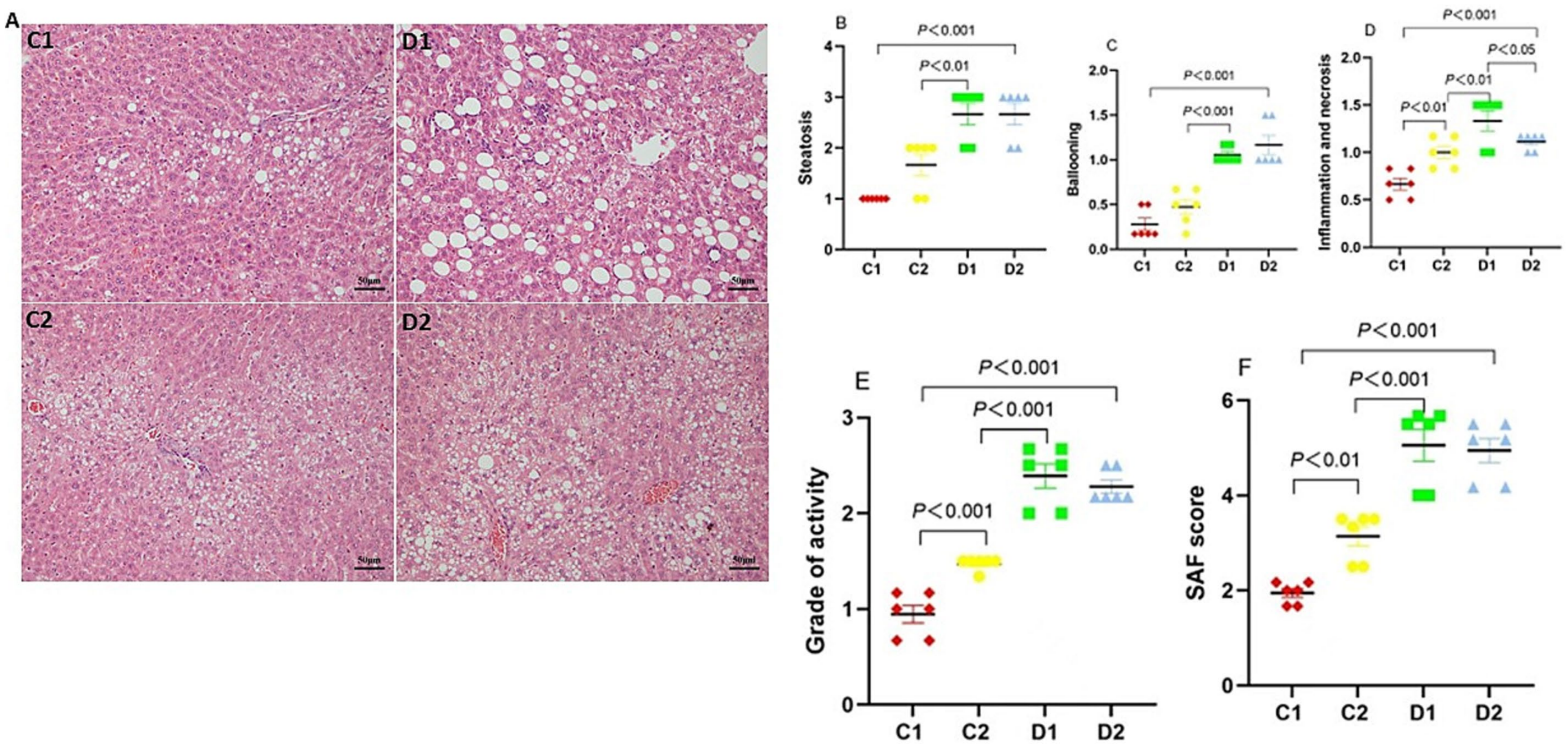
The effects of n-6 PUFA on liver histopathology in rats with NASH induced by a choline-deficient diet and. **(A)** Representative photomicrographs of liver sections stained with H&E (Scale bar – 50 μM). Refer to Supplementary Figure S1 for full-size images. **(B)** Steatosis, **(C)** Ballooning, **(D)** Inflammation and necrosis, **(E)** Grade of activity, and **(F)** SAF score. Data are expressed as mean ±SEM; *n* = 6/group.

The hallmark histopathological features of NASH, including steatosis, inflammation, and hepatocyte injury, were assessed using H&E staining (Figure 3A and see Supplementary Figure S1 for full-size images). Based on these features, we made an overall assessment of disease severity. Liver steatosis (Figure 3B) and ballooning (Figure 3C) were significantly higher in D2 vs. C1 (2.67 ± 0.21 vs. 1.00 ± 0.00, *p* < 0.001; 1.17 ± 0.11 vs. 0.28 ± 0.07, *p* < 0.001) and D1 vs. C2 (2.67 ± 0.21 vs. 1.67 ± 0.21, *p* < 0.01; 1.06 ± 0.04 vs. 0.47 ± 0.08, *p* < 0.001).

We then studied the inflammatory and necrotic changes in liver. Compared to the C1, both D2 and C2 groups exhibited increased liver inflammation and necrosis (1.11 ± 0.04 and 1.00 ± 0.06 vs. 0.67 ± 0.06, *p* < 0.001 and *p* < 0.01, respectively). Furthermore, D1 group exhibited even higher levels of liver inflammation and necrosis compared to C2 and D2 groups (1.33 ± 0.11 vs. 1.00 ± 0.06 and 1.11 ± 0.04, *p* < 0.01 and *p* < 0.05, respectively) (Figure 3D).

The grade of activity (Figure 3E) and SAF score (Figure 3F) in C2 group and D2 group were significantly higher than in C1 group (activity, 1.47 ± 0.03 and 2.28 ± 0.07 vs. 0.95 ± 0.09, both *p* < 0.001; SAF, 3.14 ± 0.20 and 4.95 ± 0.25 vs. 1.95 ± 0.09, *p* < 0.01 and *p* < 0.001, respectively); and the grade of activity and SAF score in D1 group was significantly higher than in C2 group (activity, 2.39 ± 0.13 vs. 1.47 ± 0.03, *p* < 0.001; SAF, 5.06 ± 0.34 vs. 3.14 ± 0.20, *p* < 0.001).

### Impact on hepatic lipid peroxidation, dyslipidemia and inflammatory responses

**Figure 4:**
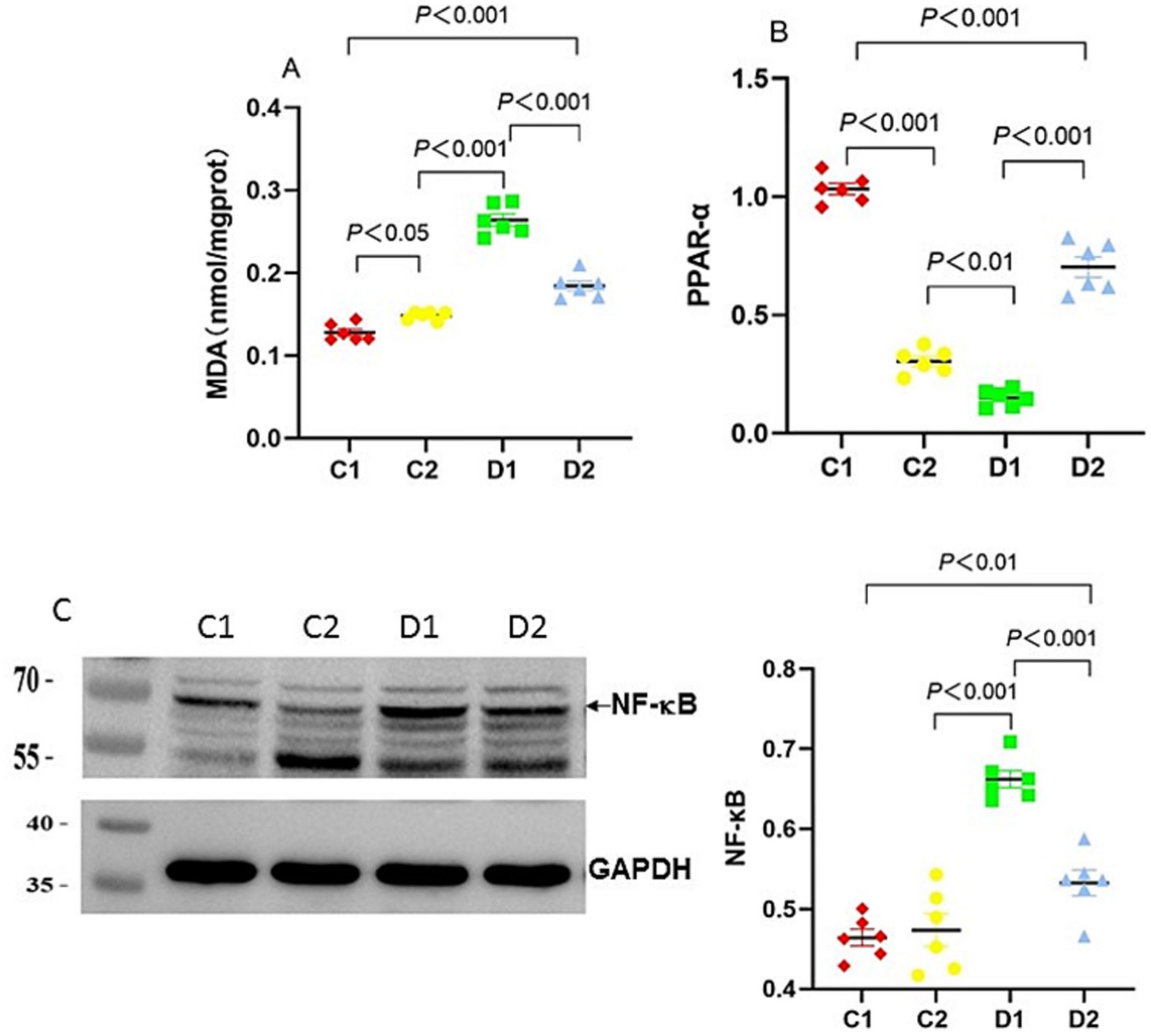
Effects of n-6 PUFA on liver lipid peroxidation and the inflammatory marker NF-κB in rats with NASH induced by a choline-deficient diet. **(A)** Liver MDA levels, **(B)** PPAR-α mRNA expression in the liver. Data are expressed as mean ±SEM; *n* = 6/group. **(C)** NF-κB protein expression (~65 kDa) in the liver as analyzed by Western blotting, normalized to GAPDH, with a representative blot (left) and quantification (right). Protein molecular weight standards (kDa) are labeled on the left of each blot. Data are expressed as mean ± SEM; *n* = 6/group.

Oxidative stress and dysregulation of transcription factors are hallmarks of NASH. We therefore assessed liver lipid peroxidation and PPAR-α and NF-κB expression to explore their potential roles. MDA (a marker of lipid peroxidation) levels in C2 and D2 were significantly higher than in C1 group (0.15 ± 0.002 and 0.18 ± 0.006 nmol/mg protein vs. 0.13 ± 0.004 nmol/mg protein, *p* < 0.05 and *p* < 0.001, respectively); MDA levels in D1 group were significantly higher than in C2 and D2 groups (0.26 ± 0.007 nmol/mg protein vs.0.15 ± 0.002 and 0.18 ± 0.006 nmol/mg protein, both *p* < 0.001) (Figure 4A).

PPAR-α is a critical regulator of lipid metabolism and fatty acid oxidation. *PPAR-α* mRNA expression in C2 and D2 groups was significantly lower than in C1 group (both *p* < 0.001). *PPAR-α* expression in D1 group was also significantly lower than in C2 (*p* < 0.01) and D2 groups (*p* < 0.001) (Figure 4B).

NF-κB levels, a key indicator of inflammation, were significantly higher in the D2 group versus the C1 group (*p* < 0.01). The NF-κB level in D1 group was also significantly higher than in C2 and D2 groups (both *p* < 0.001) (Figure 4C).

### Impact on macrophage polarization and PPAR-γ expression

**Figure 5:**
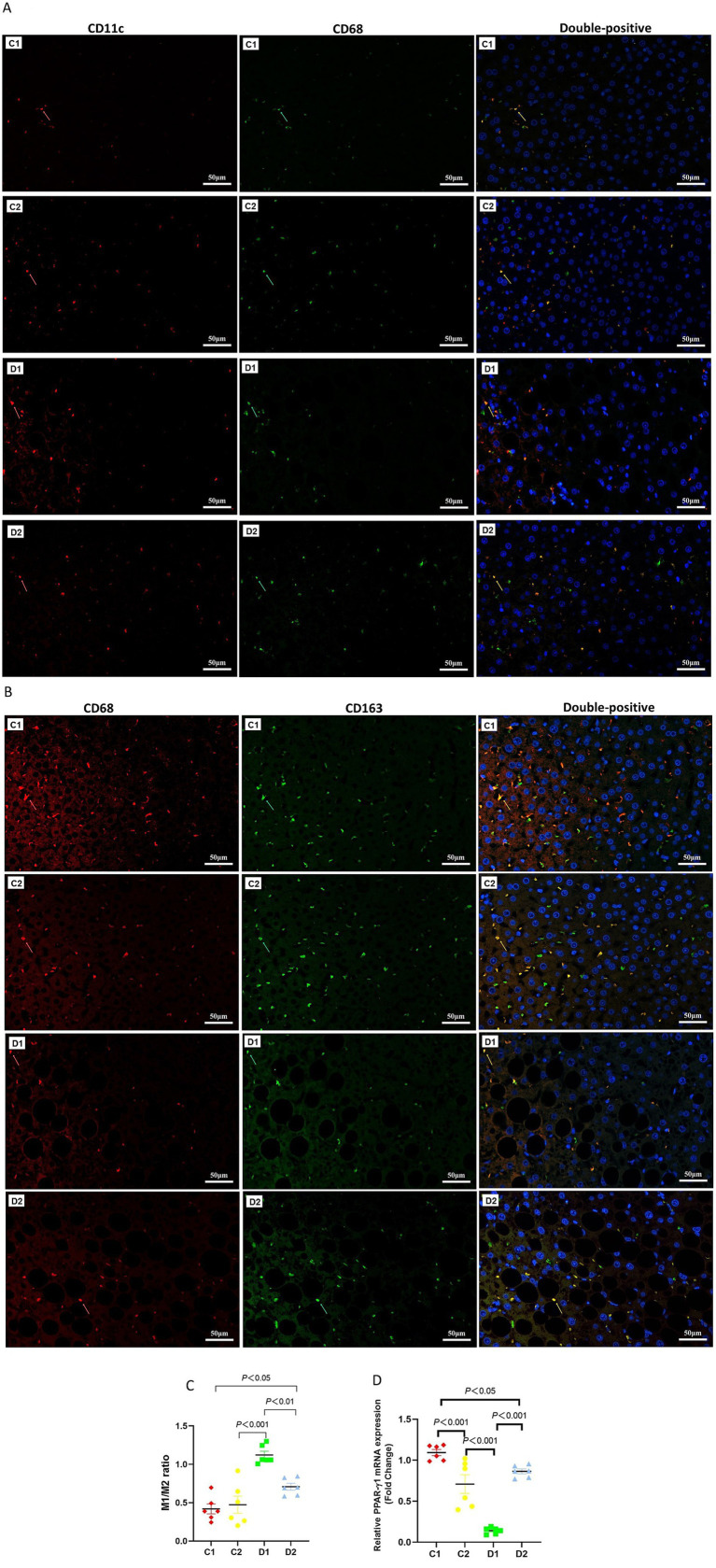
Effects of n-6 PUFA on liver macrophage phenotype in rats with NASH induced by a choline-deficient diet. **(A)** M1-type Kupffer cells (KCs) identified by double staining: red arrows show CD11c-positive cells, green arrows show CD68-positive cells, and yellow arrows highlight CD11c and CD68 double-positive M1-type KCs (Scale bar – 50 μM). **(B)** M2-type KCs identified similarly, with red arrows indicating CD163-positive cells, green arrows showing CD68-positive cells, and yellow arrows marking CD163 and CD68 double-positive M2-type KCs (Scale bar – 50 μM). For **(A,B)** (see Supplementary Figure S2) for full-size photomicrographs. **(C)** M1/M2 phenotype ratio (unitless), calculated as the proportion of CD68 + CD11c + to CD68 + CD163 + cells. **(D)** Relative *PPAR-γ2* mRNA expression (fold change normalized to GAPDH) in the liver, which is linked to macrophage polarization and inflammation. Data are expressed as mean ±SEM; *n* = 6/group.

Given NF-κB activation’s role in promoting inflammatory responses, we quantified the M1/M2 ratio, a standard measure of polarization of KCs that reflects the pro- to anti-inflammatory balance in NASH (14, 15). CD11c-positive and CD68-positive cells (yellow or orange in merged images) indicate a pro-inflammatory M1 phenotype (Figure 5A). CD68-positive and CD163-positive cells indicate an anti-inflammatory M2 phenotype (Figure 5B). Supplementary Figure S2 provides full-size images and Supplementary Table S2 shows mean counts of M1, M2, and total macrophages. The M1/M2 ratio in D2 group was significantly higher than in C1 group (0.71 ± 0.05 vs. 0.42 ± 0.07, *p* < 0.05); the M1/M2 ratio in D1 group was significantly higher than in C2 and D2 groups (1.12 ± 0.05 vs. 0.47 ± 0.11 and 0.71 ± 0.05, *p* < 0.001, *p* < 0.01, respectively).

(Figure 5C).

Given PPAR-γ’s role in promoting M2 macrophage polarization, we examined its expression in NASH livers. *PPAR-γ2* [the dominant isoform responsible for NASH progression (16, 17), 12,805,374, 17,704,301] expression in C2 and D2 groups was significantly lower than in C1 group (*p* < 0.001 and *p* < 0.05, respectively). *PPAR-γ2* expression in D1 was significantly lower than in C2 and D2 groups (both *p* < 0.001) (Figure 5D).

### Impact on hepatic cytokines profile

**Figure 6:**
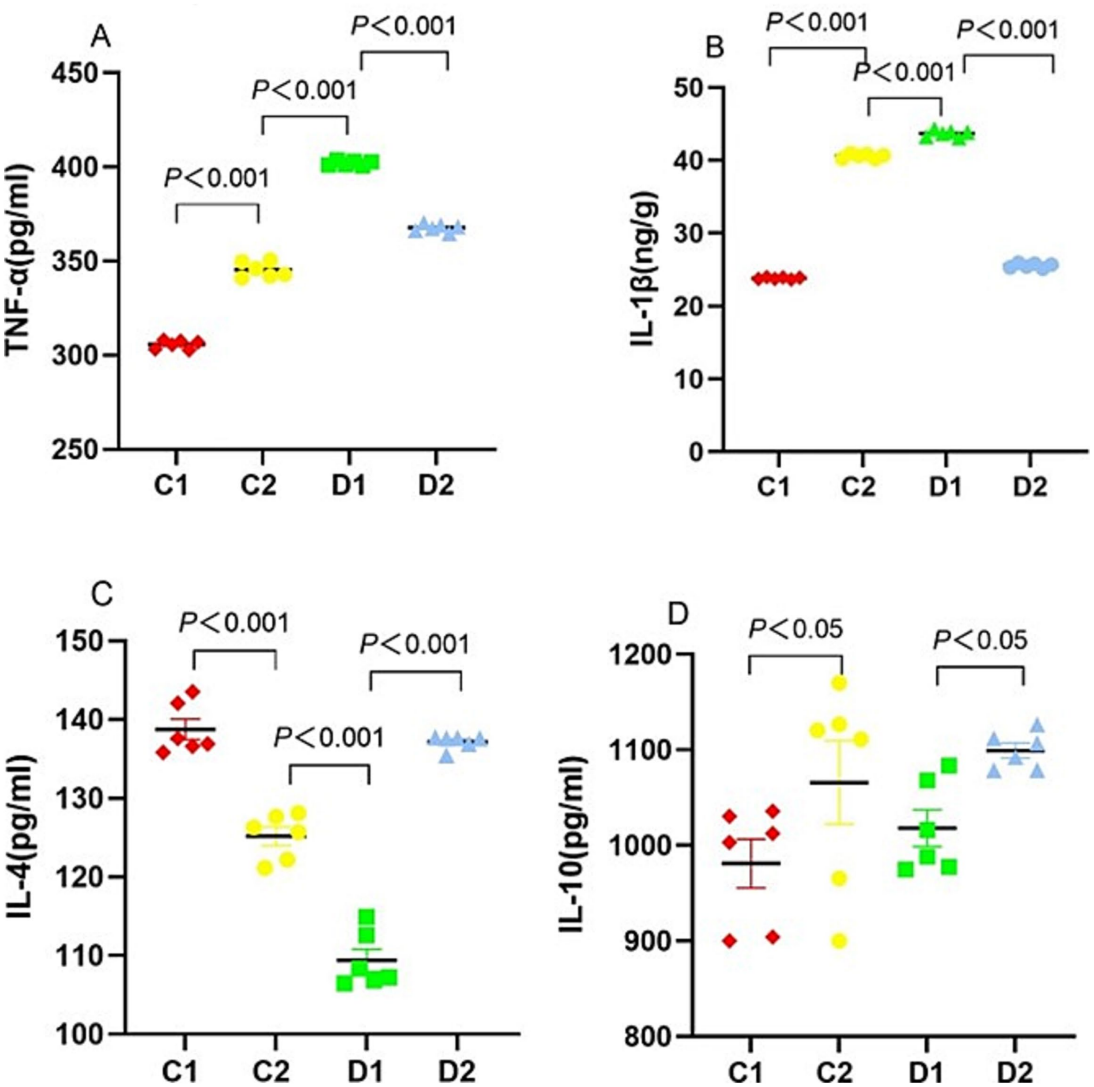
Effects of n-6 PUFA on liver cytokine levels in rats with NASH induced by a choline-deficient diet. **(A)** TNF-α and **(B)** IL-1β as pro-inflammatory cytokines; **(C)** IL-4 and **(D)** IL-10 as anti-inflammatory cytokines. Data are expressed as mean ±SEM; *n* = 6/group.

Given the role of cytokine imbalance in NASH progression, we evaluated the liver expression of TNF-α, IL-1β, IL-4, and IL-10 to elucidate the underlying pro-inflammatory and anti-inflammatory dynamics. TNF-α levels in C2 and D2 groups were higher than those in C1 group (345.42 ± 1.65 and 367.69 ± 0.84 pg./mL vs. 305.70 ± 0.93 pg./mL, both *p* < 0.001). The TNF-α level in D1 group was significantly higher than in the C2 and D2 groups (402.08 ± 0.50 pg./mL vs. 345.42 ± 1.65 and 367.69 ± 0.84 pg./mL, both *p* < 0.001) (Figure 6A).

IL-1β levels in C2 and D2 groups were significantly higher than in C1 group (40.58 ± 0.13 and 25.56 ± 0.12 pg./mL vs. 23.83 ± 0.06 pg./mL, both *p* < 0.001). The IL-1β level in D1 group was significantly higher than in C2 and D2 groups (43.69 ± 0.19 ng/g vs. 40.58 ± 0.13 and 25.56 ± 0.12 ng/g, both *p* < 0.001) (Figure 6B).

IL-4 level in C2 group was significantly lower than in C1 group (125.17 ± 1.18 pg./mL vs. 138.75 ± 1.31 pg./mL, *p* < 0.001). The IL-4 level in D1 group was significantly lower than in C2 and D2 groups (109.40 ± 1.43 pg./mL vs. 125.17 ± 1.18 and 137.16 ± 0.36 pg./mL, both *p* < 0.001) (Figure 6C). IL-10 levels were comparable across all experimental groups (Figure 6D).

### Impact on the CYP450, 5-LO, and COX-2 pathways

**Figure 7:**
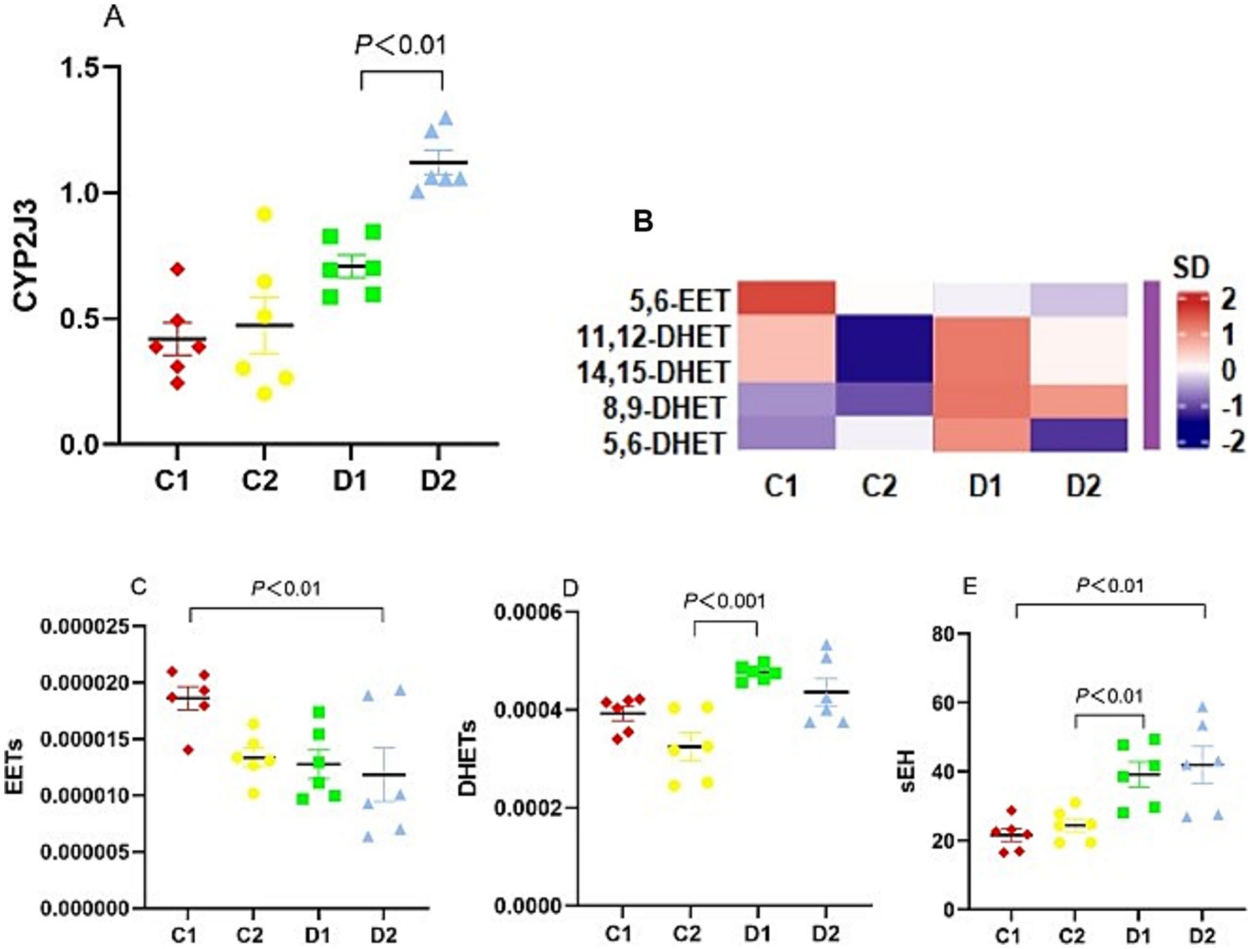
Effects of n-6 PUFA on the liver CYP450 pathway in rats with NASH induced by a choline-deficient diet. **(A)** mRNA expression of CYP450 enzyme *Cyp2j3*. **(B)** Heat map showing concentrations of CYP450 metabolites, with warm colors (red) indicating higher levels and cool colors (blue) indicating lower levels. **(C,D)** Levels of CYP450 metabolites 5,6-EET and DHETs, **(E)** sEH enzyme activity. Data are expressed as mean ±SEM; *n* = 6/group.

CYP2J3, a key enzyme in the CYP450 pathway, regulates the balance between anti-inflammatory and pro-inflammatory AA metabolites through the biosynthesis of EETs and HETEs (7). *Cyp2j3* expression in D1 group was significantly lower than in D2 group (*p <* 0.01) (Figure 7A).

To further elucidate the impact of reduced *Cyp2j3* expression on AA metabolism, we investigated the levels of EETs and DHETs using LC–MS/MS. We could detect 5,6-EET, while DHETs detected included 5,6-DHET, 8,9-DHET, 11,12-DHET, and 14,15-DHET, however, 20-HETE, a metabolite of the CYP ω-hydroxylation pathway, was not detected (Figure 7B). EET levels were significantly lower in the D2 group compared to the C1 group (*p* < 0.01) (Figure 7C), whereas DHETs levels were substantially higher in the D1 group than in the C2 group (*p* < 0.001) (Figure 7D). As sEH catalyzes the conversion of EETs to DHETs, we used the DHET-to-EET ratio as a surrogate marker for sEH activity. This analysis showed that the sEH activity in D2 group was higher than in C1 group (41.99 ± 5.33 vs. 21.60 ± 1.85, *p* < 0.01), and that in D1 group was higher than in C2 group (39.16 ± 3.64 vs. 24.45 ± 1.87, *p* < 0.01) (Figure 7E).

**Figure 8:**
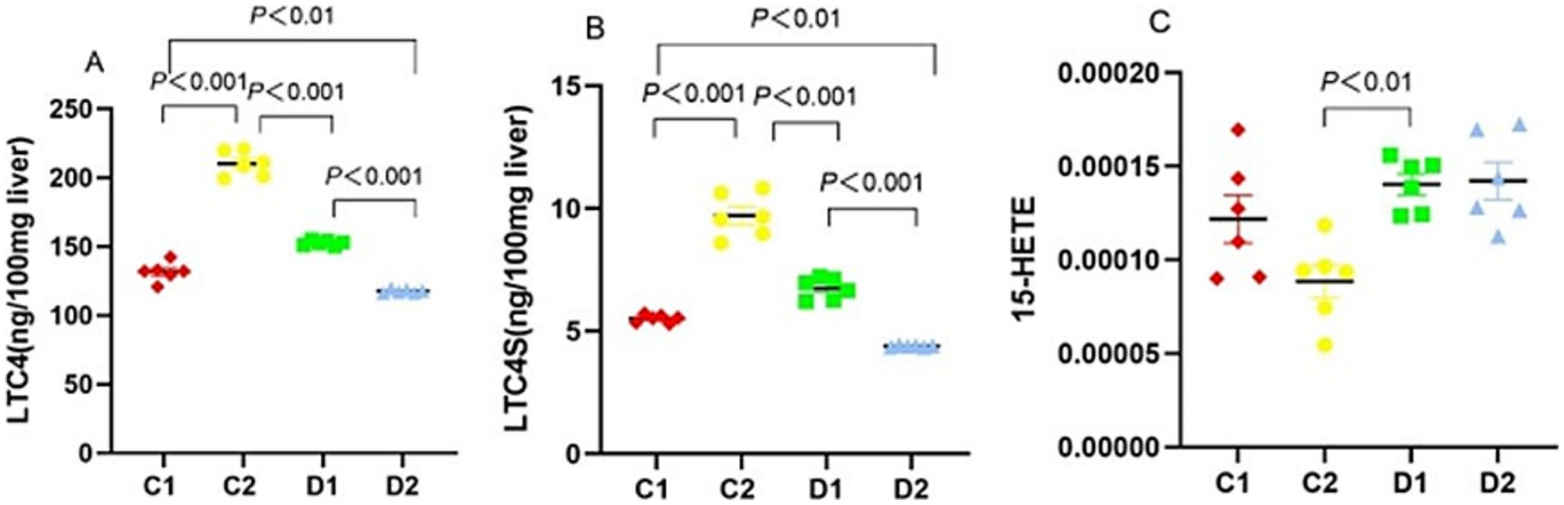
Effects of n-6 PUFA on the liver LO pathway in rats with NASH induced by a choline-deficient diet. **(A)** LTC4 and **(B)** LTC4S enzyme, and **(C)** 15-HETE. Data are expressed as mean ±SEM; *n* = 6/group.

To understand the inflammatory and oxidative stress responses in NASH, we analyzed LTC4, LTC4S, and 15-HETE, key eicosanoids derived from the LO pathway. LTC4 levels varied significantly across groups. Compared to the C1 group (131.72 ± 2.82 ng/100 mg liver), LTC4 levels were higher in the C2 group (210.27 ± 3.70 ng/100 mg liver, *p* < 0.001) and lower in the D2 group (117.87 ± 0.53 ng/100 mg liver, *p* < 0.01). In contrast, the D1 group had significantly lower LTC4 levels than the C2 group (152.71 ± 0.73 ng/100 mg liver vs. 210.27 ± 3.70 ng/100 mg liver, *p* < 0.001), but higher levels than the D2 group (*p* < 0.001) (Figure 8A). Similar to LTC4, LTC4S levels varied significantly across groups. Compared to the C1 group (5.51 ± 0.06 ng/100 mg liver), LTC4S levels were elevated in the C2 group (9.71 ± 0.36 ng/100 mg liver, *p* < 0.001) and reduced in the D2 group (4.39 ± 0.01 ng/100 mg liver, *p* < 0.01). The D1 group’s LTC4S levels (6.75 ± 0.18 ng/100 mg liver) fell between these extremes, being significantly lower than the C2 group’s and higher than the D2 group’s (both *p* < 0.001) (Figure 8B). The 15-HETE level in D1 group was higher than in C2 group (*p* < 0.01) (Figure 8C).

**Figure 9:**
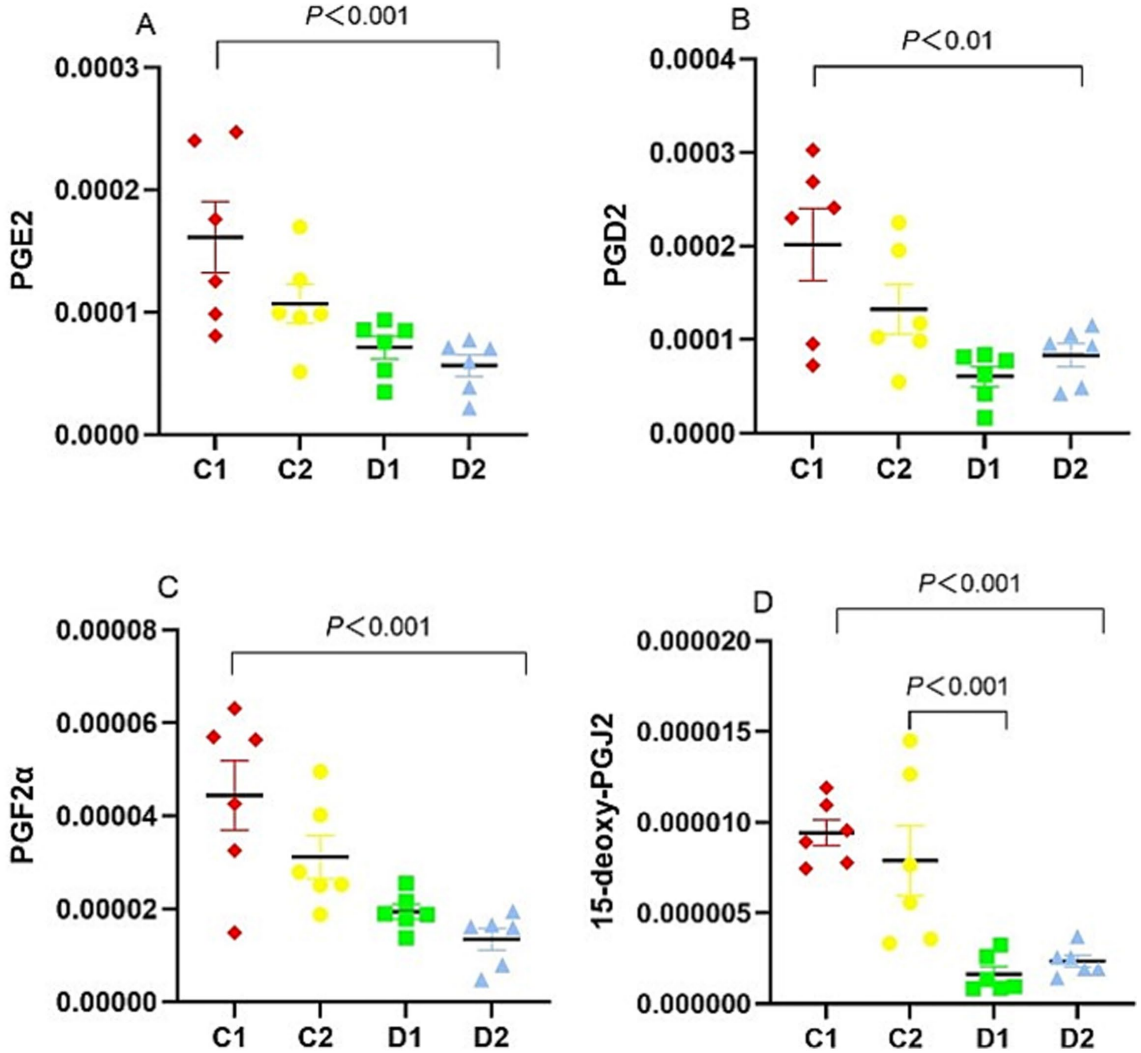
Effects of n-6 PUFA on the liver COX pathway in rats with NASH induced by a choline-deficient diet. **(A)** PGE2, **(B)** PGD2, **(C)** PGF2α, and **(D)** 15d-PGJ2. Data are expressed as mean ±SEM; *n* = 6/group.

To elucidate the role of COX-2-derived prostanoids in NASH progression, we examined the levels of PGE2, PGD2, PGF2α, and 15d-PGJ2. PGE2 (*p* < 0.001), PGD2 (*p* < 0.01), PGF_2α_ (*p* < 0.001) and 15d-PGJ2 (*p* < 0.001) levels in D2 group were significantly lower than in C1 group. Compared with C2 group, 15d-PGJ2 (*p* < 0.001) levels in D1 group were significantly lower (Figure 9).

### Interactive effects of choline deficiency and n-6 PUFA supplementation on NASH

The two-factor ANOVA revealed significant main and interaction effects between choline deficiency and n-6 PUFA supplementation (Table 3). Significant interactions between choline deficiency and n-6 PUFA supplementation were observed in several key mechanisms. Specifically, the combination of choline deficiency and n-6 PUFA supplementation exacerbated oxidative stress (MDA levels), inflammation (NF-κB, TNF-α, IL-1β, and IL-4 levels), and macrophage polarization (M1/M2 ratio). Additionally, interactive effects were seen in the regulation of PPAR-α and PPAR-γ expression, which are crucial for lipid metabolism and inflammation. Furthermore, choline deficiency and n-6 PUFA supplementation interacted to affect the grade of activity and SAF score, indicating exacerbated liver damage. These findings suggest that n-6 PUFA supplementation may amplify the detrimental effects of choline deficiency on liver health through enhanced oxidative stress, inflammation, and dysregulated lipid metabolism.

**Table 3:** Interaction effects between choline deficiency and n-6 PUFA supplementation.

Variable	Choline deficiency (*p*-value)	n-6 PUFA supplementation (*P*-value)	Interaction (*P*-value)
Liver Wet Weight	<0.01	>0.05	>0.05
Liver Index	<0.01	<0.05	>0.05
Serum AST	<0.001	>0.05	>0.05
Serum ALT	<0.01	<0.01	>0.05
Liver Steatosis	<0.001	>0.05	>0.05
Liver Ballooning	<0.001	>0.05	>0.05
Liver Inflammation/Necrosis	<0.001	<0.01	>0.05
Grade of Activity	<0.001	<0.01	<0.05
SAF Score	<0.001	<0.05	<0.05
MDA Levels	<0.001	<0.001	<0.001
PPAR-α Expression	<0.001	<0.001	<0.01
NF-κB Levels	<0.001	<0.001	<0.01
M1/M2 Ratio	<0.001	<0.01	<0.05
PPAR-γ Expression	<0.001	<0.001	<0.05
TNF-α Levels	<0.001	<0.001	<0.05
IL-1β Levels	<0.001	<0.001	<0.001
IL-4 Levels	<0.001	<0.001	<0.001
IL-10 Levels	>0.05	>0.05	<0.01
Cyp2j3 Expression	>0.05	<0.01	>0.05
EET Levels	<0.05	>0.05	>0.05
DHET Levels	<0.001	>0.05	<0.05
sEH Activity	<0.001	>0.05	>0.05
LTC4 Levels	<0.001	<0.001	<0.001
15-HETE Levels	<0.01	>0.05	>0.05
LTC4S Levels	<0.001	<0.001	<0.001
PGE2 Levels	<0.01	>0.05	>0.05
PGD2 Levels	<0.01	>0.05	>0.05
PGF2α Levels	<0.001	>0.05	>0.05
15d-PGJ2 Levels	<0.001	>0.05	>0.05

### Correlations between fatty acid metabolism pathways and inflammatory markers in NASH

Correlational analysis revealed significant associations between metabolites of the CYP450, LO, and COX-2 pathways and various markers of inflammation, lipid peroxidation, and macrophage polarization in rats with NASH induced by a choline-deficient diet (Table 4). Significant associations were observed between CYP450, LO, and COX-2 pathway metabolites and markers of inflammation, lipid peroxidation, and macrophage polarization. EET levels were positively correlated with PPAR-α expression and negatively correlated with NF-κB and TNF-α, indicating a protective role. In contrast, DHETs and sEH showed strong positive correlations with MDA, NF-κB, M1/M2 ratio, and TNF-α, indicating their role in inflammation and oxidative stress, particularly in D1 rats. Elevated 15-HETE and 15d-PGJ2 further underscored the pro-inflammatory shift, suggesting that the D1 group was most severely affected by these inflammatory and oxidative changes.

**Table 4:** Correlation between fatty acid metabolism pathways and inflammatory mediators.

Pathway	Metabolites/enzyme	Correlated marker	Correlation	*P*-value
CYP450	EETs	PPAR-α	+	*r* = 0.414, *P* < 0.05
	EETs	NF-κB	−	*r* = −0.433, *P* < 0.05
	EETs	TNF-α	−	*r* = −0.508, *P* < 0.05
	DHETs	MDA	+	*r* = 0.637, *P* < 0.01
	DHETs	NF-κB	+	*r* = 0.531, *P* < 0.01
	DHETs	M1/M2 ratio	+	*r* = 0.559, *P* < 0.001
	DHETs	TNF-α	+	*r* = 0.490, *P* < 0.05
	sEH	MDA	+	*r* = 0.521, *P* < 0.01
	sEH	NF-κB	+	*r* = 0.623, *P* < 0.01
	sEH	M1/M2 ratio	+	*r* = 0.540, *p* < 0.01
	sEH	TNF-α	+	*r* = 0.622, *P* < 0.01
LO	LTC4	IL-1β	+	*r* = 0.705, *P* < 0.001
	LTC4	PPAR-α	−	*r* = −0.593, *P* < 0.01
	15-HETE	NF-κB	+	*r* = 0.472, *P* < 0.05
	15-HETE	M1/M2 ratio	+	*r* = 0.438, *p* < 0.05
COX	PGE2	PPAR-α	+	*r* = 0.445, *P* < 0.05
	15d-PGJ2	IL-4	+	*r* = 0.432, *P* < 0.05
	15d-PGJ2	PPAR-γ	+	*r* = 0.514, *P* < 0.05
	PGE2, PGD2, PGF2α, and 15d-PGJ2	MDA	−	*r* = −0.512, *P* < 0.05 *r* = −0.456, *p* < 0.05 *r* = −0.496, *p* < 0.05 *r* = −0.674, *P* < 0.001
	PGF2α	M1/M2 ratio	−	*r* = −0.510, *P* < 0.05
	15d-PGJ2	M1/M2 ratio	−	*r* = −0.599, *P* < 0.01

(+) Positive correlation: as one variable increases, the other also increases. (−) Negative correlation: as one variable increases, the other decreases.

## Discussion

**Figure 10:**
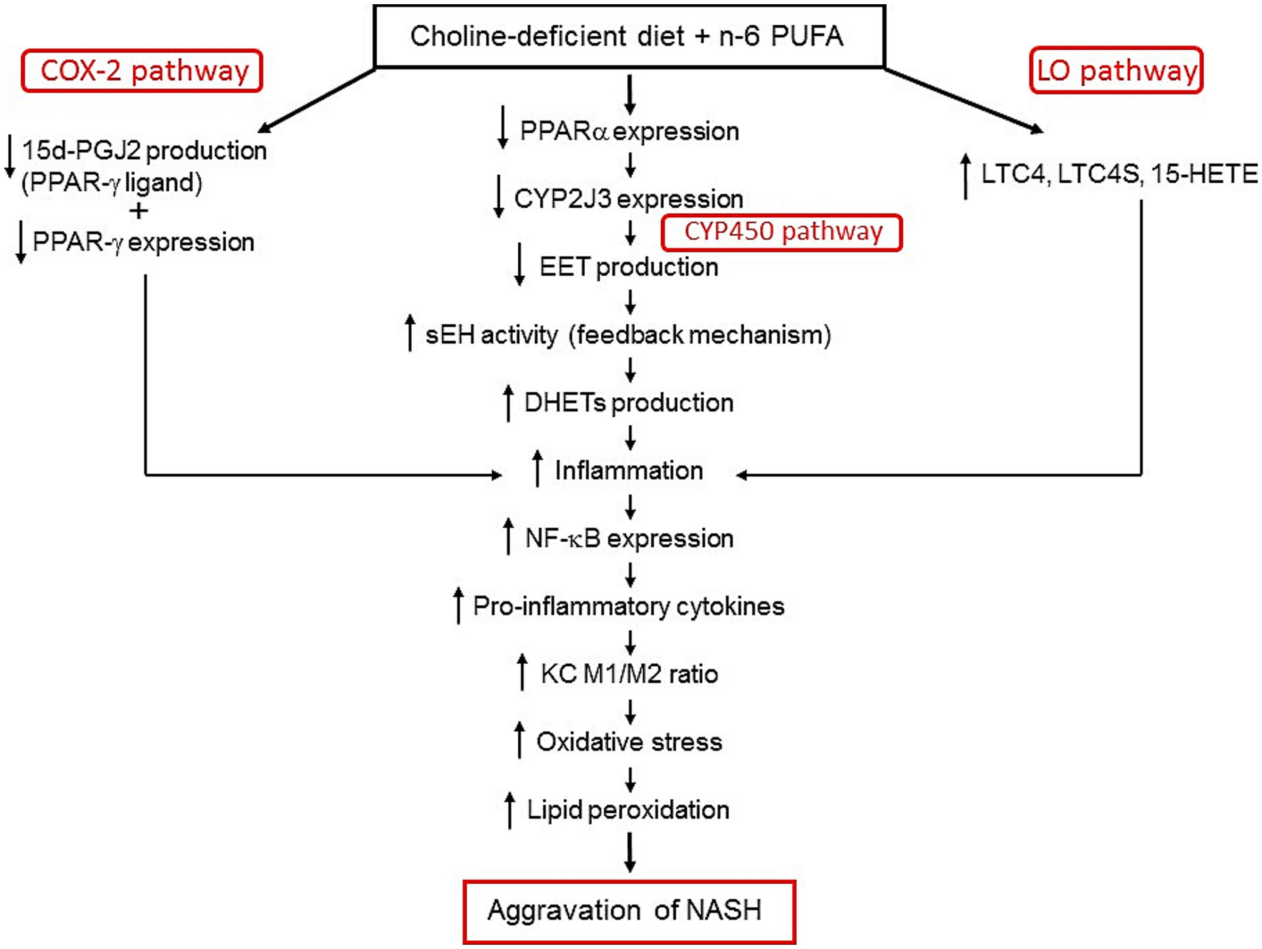
Three key pathways exacerbate NASH when n-6 PUFA is combined with a choline-deficient diet. First, the 15d-PGJ2/PPAR-γ pathway: Reduced 15d-PGJ2 decreases PPAR-γ activation, weakening anti-inflammatory effects and lipid metabolism, thereby promoting inflammation. Second, the CYP2J3/EETs pathway: Lower CYP2J3 expression decreases anti-inflammatory EETs, while increased sEH converts EETs into pro-inflammatory DHETs, further driving inflammation. Elevated DHETs also stimulate sEH activity in a feedback loop, enhancing the conversion of EETs to DHETs and worsening inflammation. Third, the LO pathway: Increased LTC4 and LTC4S (via the 5-LO pathway) and 15-HETE (via the 15-LO pathway) contribute to inflammation. Together, these pathways amplify NF-κB expression, shift cytokine balance toward a pro-inflammatory state, and promote M1 macrophage polarization over M2, leading to oxidative stress, lipid peroxidation, and hepatocyte damage.

The CDAA model, induced over 12 weeks, produces NASH-like features (steatosis, inflammation, ballooning) that mimic early-to-moderate human NASH, as supported by our studies and those of others (1–3). While it does not fully recapitulate metabolic syndrome aspects of human MASH (e.g., obesity, insulin resistance), it allows investigation of dietary impacts, such as n-6 PUFA, on liver inflammation and lipid metabolism, relevant to human dietary patterns. This study demonstrates how high n-6 PUFA, in combination with choline deficiency, worsens NASH through distinct metabolic pathways (summarized in Figure 10). The 15d-PGJ2/PPAR-γ pathway, which reduces 15d-PGJ2 lowers PPAR-γ activation, weakening anti-inflammatory and lipid metabolism effects, increasing pro-inflammatory cytokines and suppressing anti-inflammatory cytokines. In the CYP2J3/EET pathway, reduced CYP2J3 expression limits anti-inflammatory EET production, while elevated sEH drives the conversion of EETs to pro-inflammatory DHETs, amplifying inflammation and oxidative stress. The LTC4/LO pathway, with elevated LTC4 and 15-HETE, further disrupts cytokine balance and inflammation. The combined effect of these three metabolic pathways alteration leads to imbalances in cytokines levels, increase inflammation leading to higher levels of M1 macrophage in liver of NASH rats exacerbating liver damage by lipid peroxidation evident from increased MDA levels. The integration of correlation analysis to link these pathways with specific inflammatory and oxidative markers provides a novel perspective on the metabolic interactions driving NASH progression.

We evaluated steatosis using H&E staining and SAF scoring, which confirmed lipid accumulation in the CDAA-induced NASH model. However, additional lipid-specific staining like Oil Red O could have further validated hepatic lipid accumulation. This study focused on oxidative stress and inflammation, as lipid peroxidation and oxidative stress facilitate the progression of NAFLD to NASH (7). Lipid peroxidation, marked by elevated MDA is a key marker of oxidative stress, while PPAR-α regulates lipid homeostasis and inflammation (18, 19). Studies show that a MCD diet decreases PPAR-α and increases MDA, leading to liver lipid peroxidation (13). In our study, feeding a choline-deficient diet lowered PPAR-α and raised MDA levels in NASH, with stronger effects in rats supplemented with n-6 PUFA than those with SFA. Liver inflammation leading to NASH is strongly mediated by NF-κB and the M1/M2 KC phenotype ratio (20, 21). Pro-inflammatory M1 KCs secrete TNF-α and IL-1β, while anti-inflammatory M2 KCs secrete IL-4 and IL-10. PPAR-γ promotes M2 polarization and reduces inflammation by inhibiting NF-κB κB (22–24). Reports show that MCD and CDAA diets increase NF-κB, TNF-α, IL-1β, and decrease IL-4 and PPAR-γ expression, worsening inflammation (25–27). Our findings advance these reports by showing that a choline-deficient diet with low n-6 PUFA (D2 group) decreases PPAR-γ and increases NF-κB, TNF-α, IL-1β, and the M1/M2 ratio, while reducing IL-4 in NASH livers, with choline deficient diet with high n-6 PUFA (D1 group) further exacerbating these inflammatory changes.

The CYP450 pathway, specifically the CYP2J family, plays a key role in liver inflammation, with CYP2J3 as the predominant form in rats and mice (28). CYP2J3 overexpression has been reported to increase EET levels in diabetic mice to ameliorate insulin resistance (28) EETs are generally decreased in NASH induced by high-fat diets, while their levels increase with MCD (29–32). There are reports of both increased and decreased EET levels in NASH patients (33). In our study, the D2 group showed a significant reduction in hepatic EET levels, while levels in the D1 group were comparable to controls, indicating that EET levels may not be reliably associated with NASH progression. However, correlation analyses revealed EETs were positively linked to PPAR-α and negatively to NF-κB and TNF-α, suggesting that EETs may have anti-inflammatory and hepatic lipid regulatory functions. Although DHET was previously regarded as an inactive metabolite, recent findings suggest it plays a detrimental role in metabolism, contributing to inflammation and disrupting metabolic processes (31, 34–36). We observed significantly higher DHET levels in rats supplemented with n-6 PUFA, and these levels were positively correlated with pro-inflammatory cytokines, highlighting DHET’s emerging role as a pro-inflammatory and metabolically detrimental factor.

sHE catalyzes the conversion of EETs to DHETs, making sEH inhibitors potential treatments for non-alcoholic fatty liver disease (NAFLD) by decreasing lipid peroxidation, inflammation, and fibrosis (37). In our study, both D1 and D2 groups had higher sHE levels than the other two groups. Correlation analyses revealed that DHET and sEH were linked to increased MDA, NF-κB, and inflammation. Our data further revealed that NASH exacerbation in the D1 group is driven by increased DHETs, indicating heightened sEH activity that converts protective EETs into inflammatory DHETs. While EETs were decreased in D2, DHETs were not elevated, suggesting that increased DHET levels in the D1 group were driving the exacerbation of NASH.

In addition to increased sEH activity converting protective EETs into inflammatory DHETs, which worsens inflammation in NASH, elevated 15-LO activity enhances 15-HETE production in some NASH patients (10, 38) further exacerbating liver inflammation and disease progression. Plasma 15-HETE levels in patients with severe NAFLD are non-significantly elevated (39). In our study, the increase in 15-HETE along with LTC4 and LTC4S (resulting from 5-LO activity) indicate that high n-6 PUFA (in D1 group) exacerbates inflammation and lipid peroxidation in a choline-deficient state via the LO pathway. However, elevated 15-HETE in D1 compared to C2 (*p* < 0.01) reflects the effect of choline deficiency, with no significant n-6 PUFA effect or interaction (Table 3).

The COX pathway, involved in NASH inflammation, metabolizes AA into metabolites including PGE2, PGD2, and TXA2 (40)^.^ In mice with MCD diet-induced NASH, COX-2 levels are increased in the liver, activating NF-κB and upregulating TNF-α and IL-6 (41)^.^ Although, the inflammatory role of some prostaglandins remains controversial (40), however, PGD2 and its metabolite 15d-PGJ2 play anti-inflammatory roles (6, 40, 42). 15d-PGJ2 is a natural ligand of PPAR-γ, and inhibits NF-κB and other inflammatory signaling pathways through PPAR-γ-dependent and independent mechanisms, and is involved in the regulation of chronic inflammation (43). We observed lowered hepatic levels of PGE2, PGD2, PGF2α, and 15d-PGJ2 in D2 group, thereby correlating choline deficiency negatively with MDA, NF-κB, and TNF-α, suggesting anti-inflammatory effects through the COX-2 pathway. Lower 15d-PGJ2 levels in D2 compared to C1 (*p* < 0.001) and further reduction in D1 compared to C2 (p < 0.001) suggest that CDAA primarily drives reduced anti-inflammatory 15d-PGJ2, with n-6 PUFA having no significant independent effect (Table 3). The increased use of n-6 PUFA for cardiovascular health may raise the risk of NASH (37). We observed that choline-deficient diet with high n-6 PUFA (D1 group) exacerbates NASH more severely than choline deficiency alone (D2 group) through multiple pathways. In D1 group, there is a significant increase in MDA levels, indicating enhanced lipid peroxidation and oxidative stress. This is accompanied by elevated NF-κB and TNF-α, further driving inflammation. Additionally, D1 group shows reduced PPAR-α expression, which impairs the liver’s ability to regulate fatty acid metabolism and inflammation. The diet also increases DHETs and LTC4, while decreasing protective prostaglandins like 15d-PGJ2 and TXB2. Together, these changes contribute to a more pronounced inflammatory response and accelerated progression of NASH in the D1 group compared to D2.

## Conclusion

In this study, we elucidated multiple mechanisms by which n-6 PUFA exacerbates liver lipid peroxidation and inflammation in NASH, highlighting the unique interplay between n-6 PUFA and choline deficiency (Figure 10). Our findings provide new insights into how n-6 PUFA accelerates NASH progression through specific biochemical pathways.

## Data Availability

The original contributions presented in the study are included in the article/Supplementary material, further inquiries can be directed to the corresponding authors.
