# Supplementary material for: High intake of n-6 polyunsaturated fatty acid exacerbates non-alcoholic steatohepatitis by the involvement of multiple metabolic pathways
Source: Front Nutr. 2025 Jun 23;12:1562509. doi: 10.3389/fnut.2025.1562509 (PMC12229839; doi:10.3389/fnut.2025.1562509)
Supplement: Supplementary file 1 [file Table_1.docx]

**Supplemental Table S1**. Nutritional composition of diets used in the study.

| **Composition** | **C1 (CSAA + low n-6 PUFA)** | **C2 (CSAA + high n-6 PUFA)** | **D1 (CDAA + high n-6 PUFA)** | **D2 (CDAA + low n-6 PUFA)** |
| --- | --- | --- | --- | --- |
| **Macronutrients (% by weight)** | | | | |
| Protein | 20.0 | 20.0 | 20.0 | 20.0 |
| Fat | 10.0 | 10.0 | 10.0 | 10.0 |
| Carbohydrate | 63.0 | 63.0 | 63.0 | 63.0 |
| Fiber | 5.0 | 5.0 | 5.0 | 5.0 |
| **Key Micronutrients (g/kg)** | | | | |
| Vitamin Mix (AIN-93M) | 10.0 | 10.0 | 10.0 | 10.0 |
| Mineral Mix (AIN-93M) | 35.0 | 35.0 | 35.0 | 35.0 |
| **Other ingredients (g/kg)** | | | | |
| L-Amino Acid Mix (AIN 93M)* | 200.0 | 200.0 | 200.0 | 200.0 |
| Corn Starch | 397.5 | 397.5 | 397.5 | 397.5 |
| Dextrinized Corn Starch | 132.0 | 132.0 | 132.0 | 132.0 |
| Sucrose | 100.0 | 100.0 | 100.0 | 100.0 |
| Cellulose | 50.0 | 50.0 | 50.0 | 50.0 |
| Mineral Mix (AIN-93M) | 35.0 | 35.0 | 35.0 | 35.0 |
| Vitamin Mix (AIN-93M) | 10.0 | 10.0 | 10.0 | 10.0 |
| Antioxidant (Tertiary-Butylhydroquinone) | 0.02 | 0.02 | 0.02 | 0.02 |
| **Experimental group-specific ingredients (g/kg)** | | | | |
| Choline chloride | 3.0 | 3.0 | 0.0 | 0.0 |
| n-6 PUFA (Linoleic Acid) | 3.0 | 57.0 | 57.0 | 3.0 |
| Coconut Oil | 100.0 (equivalent to 10g per 100g) | 0.0 | 100.0 (equivalent to 10g per 100g) | 0.0 |
| Corn Oil | 0.0 | 100.0 (equivalent to 10g per 100g) | 0.0 | 100.0 (equivalent to 10g per 100g) |
| **Energy density (kcal/g)** | 4.2 | 4.2 | 4.2 | 4.2 |

TP 01010GS, Cat # for CSAA diet and TP 01010G, Cat# for CDAA diet; source, Nantong Trophic Feed Technology Co., Ltd., China.

* Follows rodent AIN-93M diet proportions, providing 20% protein equivalent, with no choline-containing compounds.

Energy calculated as: 4 kcal/g (protein, carbohydrate), 9 kcal/g (fat), excluding fiber.
