# Supplementary material for: High intake of n-6 polyunsaturated fatty acid exacerbates non-alcoholic steatohepatitis by the involvement of multiple metabolic pathways
Source: Front Nutr. 2025 Jun 23;12:1562509. doi: 10.3389/fnut.2025.1562509 (PMC12229839; doi:10.3389/fnut.2025.1562509)
Supplement: Supplementary file 2 [file Table_2.docx]

**Supplementary Table 2**. Counts of M1, M2, and total macrophages (KCs) in NASH liver sections

| **Groups** | **M1 (CD68+CD11c+) counts (Mean ± SEM)** | **M2 (CD68+CD163+) counts (mean ± SEM)** | **CD68 (total macrophages) counts (mean ± SEM)** |
| --- | --- | --- | --- |
| C1 | 18.83 ± 1.32 | 45.11 ± 2.24 | 131.06 ± 4.77 |
| C2 | 21.50 ± 2.92 | 44.06 ± 1.41 | 124.61 ± 6.51 |
| D1 | 38.11 ± 1.04 | 38.39 ± 1.27 | 70.5 ± 3.09 |
| D2 | 31.4 ± 1.6 | 41.6 ± 2.0 | 108.9 ± 4.4 |
